# Informing Disease Models with Temporal and Spatial Contact Structure among GPS-Collared Individuals in Wild Populations

**DOI:** 10.1371/journal.pone.0084368

**Published:** 2014-01-07

**Authors:** David M. Williams, Amy C. Dechen Quinn, William F. Porter

**Affiliations:** Department of Environmental and Forest Biology, State University of New York College of Environmental Science and Forestry, Syracuse, New York, United States of America; Umeå University, Sweden

## Abstract

Contacts between hosts are essential for transmission of many infectious agents. Understanding how contacts, and thus transmission rates, occur in space and time is critical to effectively responding to disease outbreaks in free-ranging animal populations. Contacts between animals in the wild are often difficult to observe or measure directly. Instead, one must infer contacts from metrics such as proximity in space and time. Our objective was to examine how contacts between white-tailed deer (*Odocoileus virginianus*) vary in space and among seasons. We used GPS movement data from 71 deer in central New York State to quantify potential direct contacts between deer and indirect overlap in space use across time and space. Daily probabilities of direct contact decreased from winter (0.05–0.14), to low levels post-parturition through summer (0.00–0.02), and increased during the rut to winter levels. The cumulative distribution for the spatial structure of direct and indirect contact probabilities around a hypothetical point of occurrence increased rapidly with distance for deer pairs separated by 1,000 m – 7,000 m. Ninety-five percent of the probabilities of direct contact occurred among deer pairs within 8,500 m of one another, and 99% within 10,900 m. Probabilities of indirect contact accumulated across greater spatial extents: 95% at 11,900 m and 99% at 49,000 m. Contacts were spatially consistent across seasons, indicating that although contact rates differ seasonally, they occur proportionally across similar landscape extents. Distributions of contact probabilities across space can inform management decisions for assessing risk and allocating resources in response.

## Introduction

Understanding the temporal and spatial structure of direct and indirect contacts among individuals or groups within a population is useful for attempting to control or eradicate disease [1], [2], [3]. Here we use white-tailed deer (*Odocoileus virginianus*) tagged with global positioning system (GPS) collars to investigate how the likelihood of putative contacts changes with time and distance between individuals. Our primary motivation is to understand the influence of contact rates on the risk of spread of chronic wasting disease (CWD) among deer in central New York. The approach and findings, however, lend themselves to more general consideration of estimating how contacts vary in time and across space.

Infectious diseases require contacts between hosts, hosts and vectors, or hosts and infectious propagules for transmission. Understanding contacts among individuals or groups within a population is useful for protecting and managing populations threatened by disease and in attempting to control or eradicate disease [1], [2], [4]. When an infectious disease is introduced into an animal population, public officials and scientists must decide whether to respond, and if so, how and where to allocate limited resources in response to points of disease outbreaks. When a disease is detected at a given location, 2 of the most important questions are, how far could it spread within a given period of time or where else might it be? These became key questions in New York State in 2005 when 2 free-ranging deer tested positive for chronic wasting disease (CWD). Without empirical data, the decisions of how and where to respond were based upon the feasibility of management within political units, the dispersion of CWD in established areas outside of New York, and the general biology associated with ranging behavior of white-tailed deer. A containment area was established to serve as a regulatory boundary with the intent of preventing the spread and export of the infection.

Responses to disease outbreaks may be refined by simulation models that explore the range of potential outcomes for disease dynamics, the most influential components of disease spread, and what information is lacking. Central to these models is the transmission parameter, *β*, the probability of transmission given the disease is encountered. However, the transmission parameter is the most difficult coefficient to empirically estimate [5]. Transmission may be estimated by fitting a given model to temporal prevalence data [6]. This approach depends upon assumptions about the probability of infection given a contact (density or frequency dependent), and contact rate per se is often not distinguished as a separate entity [3]. Emerging infectious diseases where only a few individuals are infected such as CWD in New York lack the prevalence data required for such calculations and require alternative means for estimating the transmission coefficient.

When attempting to inform our understanding of transmission using empirical data, it is useful to consider transmission as a 2-part process: 1) the probability of an infection given a contact and, 2) the rate of appropriate contacts. The first component, the probability of infection given a contact, may be informed by captive control studies where individuals are intentionally exposed to the disease in question. However, the limited understanding of prion infections such as CWD and the protracted incubation period of CWD make such estimates both expensive and difficult. Here, we focus on understanding the second component of transmission, the probability that contact occurs.

One alternative for estimating contact rates in the absence of sufficient prevalence data is to use existing information about host behavior and disease biology [5]. Many animals, even humans, exhibit highly seasonal behaviors. The degree to which contact events change seasonally has important implications for potential spread of disease. Temporal variability of contact rates, particularly within host aggregations, is an important driver of measles, mumps, chicken pox, and influenza dynamics among humans [3]. In their study of mycoplasmal conjunctivitis among house finches (*Carpodacus mexicanus*), Hosseini et al. [7] concluded that seasonal host behaviors could be of primary importance in understanding wildlife disease dynamics. Tasmanian devils (*Sarcophilus harrisi*) exhibited seasonal variation in incidence of bite injuries [8] and contact structure [9]. Authors concluded that both of these factors likely contributed to the dynamics of devil facial tumor disease. Likewise, the behavior of white-tailed deer varies seasonally with activities such as winter migration, breeding, and parturition. These behaviors lead to different population densities, movement rates, and contacts between individuals [10], [11], [12]. These variations may seasonally alter the transmission of a disease and result in complex disease dynamics [3], [13]. Grassly and Fraser [14] describe potential impacts of seasonal variation in transmission on disease dynamics including a fluctuating basic reproductive number (R_0_), larger outbreaks, large oscillations in incidence, and reduced persistence. Potential transmission by indirect contact with environmental contaminates (e.g., shed CWD prions) may also vary seasonally and further complicate potential disease dynamics not only as another mode of transmission, but also by introducing time lags.

Contacts between animals have been measured using a number of techniques including direct observation of animals [8], [15], [16], overlap of landscape usage [12], [17], [18], separation distance of telemetry locations [12], [19], [20], [21], and proximity collars [9], [22], [23]. Direct observations are particularly useful because they enable identification of probable contacts for the disease in question, but are limited to short periods of observation and may depend upon unnatural conditions (such as contact around baiting stations). Identifying areas of joint space use is relatively easy provided sufficient animal location data are available. Intuitively, greater overlap in space use would translate into increased potential for contacts, but this assumes that animals move around and contact one another randomly. Proximity collars can record when and how long collars are within a certain distance and come closest to identifying actual direct contact events (i.e., synchronous spatial overlap), but unless coupled with recorded positions through time are unable to inform rates of indirect contact (i.e., asynchronous spatial overlap) between individuals. Battery life may be quickly exhausted in proximity collars if individuals remain in close proximity for extended periods of time [22]. Data from GPS collars are limited to points in time, but locations may be collected frequently and systematically over long periods with little positional error. The widespread availability and use of GPS animal-tracking devices and their potential to identify both direct and indirect contact events make them useful for quantifying contact events in wildlife populations.

CWD among deer is an excellent system to evaluate potential contact events to inform aspects of transmission for several reasons. First, CWD is known to be transmitted directly via physical interactions between conspecifics as well as indirectly when an individual encounters an area of landscape where infectious prions have previously been shed by another individual through saliva, feces, urine, or decomposing carcass [24], [25]. Prions can persist in soil for long periods of time and sites can remain contaminated for years [24]. However, the relative importance of these direct and indirect routes is unknown. Second, white-tailed deer exhibit season specific movements, social structuring, and behaviors allowing for both direct and indirect routes of transmission. These behaviors suggest we should expect contact rates to vary in time and space. Finally, while prevalence data are generally insufficient or too coarse to evaluate variation in contact rates empirically, deer movement can be reasonably described using GPS collars. Deer are readily captured, large enough to accommodate GPS collars with sufficient battery life to monitor for extended periods of time, and move at scales that are large relative to the positional error of those collars.

Previous research has shown variation in deer space use and scales of movement among seasons, sexes, and age classes. We sought to estimate probabilities of direct contact between individuals and indirect contact between an individual and an environment previously occupied by other individuals as functions of space and time. We drew on a large sample of white-tailed deer tagged with GPS collars in central New York. Our objectives were to quantify the rate that the probability of contact diminishes with distance between individuals and identify the seasonal dynamics of contact events. We hypothesized that contacts would be most likely when deer aggregate for feeding and breeding, and so the probabilities of contact would reflect seasonal changes in deer behavior. Specifically we expected contacts to be most frequent during winter when deer migrate to specific habitat cover types in response to snowfall, and contacts to be least frequent during fawning when does isolate themselves. We also predicted that, due to potential social structure among individuals, the probability of deer contacting one another would decrease rapidly with increasing separation distance.

## Methods

This study utilized data from GPS collared deer which were captured, collared, and monitored according to and with approval by State University of New York College of Environmental Science and Forestry Institutional Animal Care and Use Protocol no. 2005-1.

This study took place on both public and private lands. We received permission from the New York Department of Environmental Conservation and private landowners for all capture locations and collar retrievals on public and private lands respectively. We were not required to obtain permission for lands used by free-ranging collared animals where we were not physically engaged in research activities.

### Study area

The study area encompassed 8,300 km^2^ in Onondaga, Cortland, Madison and Oneida Counties of central New York State. Landcover was a mix of forest (44%) and agriculture (34%) with small communities (9% developed). Forests were dominated by hardwoods, notably sugar and red maple (*Acer saccharum* and *A. rubrum*), American beech (*Fagus grandifolia*), white ash (*Fraxinus americana*) and black cherry (*Prunus serotina*). Conifer plantations originating in the 1930’s were composed of white, red and Scotch pine (*Pinus strobus, P. resinosa, and P. sylvestris*), and white and red spruce (*Picea glauca, P. rubens*). Agricultural crops were mostly related to dairy and include corn, winter wheat, oats, alfalfa, and soybeans. A rolling topography occurred throughout those portions of the study area in Onondaga, Cortland and Madison Counties; areas in Oneida County occur on glacial lake plain. Average temperatures were −5.0°C during February and 20.6°C in July (1966–2006). Elevations range from 93 m to 652 m and the region lies to the south and east of Lake Ontario. The combination of the prevailing wind patterns and elevation affects precipitation. Average total annual precipitation was 97.3 cm/year (1966–2006). Winters are variable with heavy snow events and frequent thaws. Snowfall averaged 251 cm/year (1966–2006) and ranged from 241 cm/yr to 336 cm/yr during this study [26]. The deepest snowpack (74 cm) during our study occurred in Oneida County in February of 2007 [26]. Road density in the region was 1.85km/km^2^; 1.5% of the landscape was >1.6km from a road [27].

### Movement data

We used data from GPS collars (model GPS2000, Advanced Telemetry Systems, Inc.) on 71 white-tailed deer (27 males and 44 females), captured during January-April 2006 and 2007 using modified Clover traps [28], rocket nets, and dart guns (see Dechen Quinn et al. [29] for capture and handling details). Deer were captured and collared individually with the exception of 3 pairs. We never captured more than 2 deer per trapping event. Collars were programmed to take a GPS location every 5 hr. GPS locations were stored on board the collars that were remotely detached from study animals and retrieved after approximately 1 yr (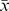 = 271 days). Positional error associated with GPS locations was <10m in most cases (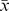 = 5.3 m, SD = 5.3 m) [30].

### Defining contact events

Because GPS collars do not record positions continuously, we could not observe contacts directly. Rather, we assume that animals close in space are likely or at least have the opportunity to come into contact with one another. We used the 5 hr GPS location data to determine probabilities of direct and indirect contact as functions of time and space. We measured the distance between pairs of animals at synchronous locations in a similar manner as Schauber et al. [12]. Not all collars acquired positional fixes on the same schedule so we designated a time window or lag during which to associate points in time. We divided days into six 4-hr periods and locations for a pair of individuals that occurred within the same 4-hr window were considered direct contacts. We assumed that animals within 100 m of one another during a synchronous time period did, or could have, contacted. We then counted the number of contacts between all pairs of collared deer in each interval. We pooled contact events of all deer pairings by day of year to explore the temporal structure of direct and indirect contacts. We calculated daily probabilities of contact by dividing the number of daily contact events by the total number of synchronous locations observed that day. We also evaluated cutoff distances from 25 to 500 m to assess the sensitivity of subsequent analyses to this contact criterion.

We defined indirect contacts as observed locations of a deer that occurred within 100 m of any position previously occupied by another individual. For example, at each of deer A's locations we evaluated whether deer B occupied a location ≤100 m at the same or previous time. If so, deer A's position was recorded as an indirect contact regardless of the number of times deer B previously occupied space within 100 m. Subsequent visits by deer A to that position would be recorded as additional indirect contacts. Our estimate of the probability of indirect contact for each deer pair was calculated by dividing the number of indirect contacts by the total number of observations where previously recorded positions were observed.

### Temporal contact structure

We evaluated how contacts change throughout the year by modeling contact probability as a function of day of year. We considered sine functions of varying complexity, including combinations of parameters controlling the amplitude, phase shift, and intercept. We also evaluated piecewise linear threshold models representing 3 and 4 season years (Table 1). We used Akaike's Information Criterion (AIC) to select the best model and quantify the degree to which those functions differed from one another and from a null model representing the hypothesis that contact rates do not vary with time (intercept only). Model fitting and evaluation were conducted in R v2.90 [31]. We conducted k-fold cross validation (k = 5) and goodness-of-fit tests (based on 20% quantile bins) to evaluate the predictive accuracy of the best models. Qualitative comparisons were also conducted to verify correspondence between observed contact dynamics and well-documented seasonal deer ecology and behavior.

**Table 1:** Model comparisons describing temporal dynamics of daily contact probability among deer pairs separated by <11 km in central New York.

Contact	Model	Breakpoints (YDAY)	*a* (SE)	*b* (SE)	*c* (SE)	ΔAIC	*w*_i_
*Direct*	p(contact) ∼*a*+*b* * sin((YDAY+*c*) * (π/182.5))		0.037 (8.8E-04)	0.033 (0.001)	−130 (2.20)	0	0.999
	threshold - piecewise linear - 4 breakpoints	87, 147, 311, 356	-	-	-	13.59	0.001
	threshold - piecewise linear - 3 breakpoints	116, 311, 365	-	-	-	71.20	3.46E-16
	p(contact) ∼*a*+*b* * sin((YDAY) * (π/182.5))		0.037 (0.001)	0.020 (0.002)	-	287.29	4.12E-63
	p(contact) ∼*a*		0.037 (0.001)	-	-	383.87	4.40E-84
*Indirect*	threshold - piecewise linear - 4 breakpoints	84, 150, 280, 365	-	-	-	0	0.999
	threshold - piecewise linear - 3 breakpoints	84, 140,365	-	-	-	22.32	1.42E-05
	p(contact) ∼*a*+*b* * sin((YDAY+*c*) * (π/182.5))		0.133 (0.003)	-0.028 (0.001)	−140 (3.01)	153.61	4.41E-34
	p(contact) ∼*a*+*b* * sin((YDAY) * (π/182.5))		0.133 (0.001)	0.021 (0.002)	-	288.51	2.25E-63
	p(contact) ∼*a*		0.133 (0.001)	-	-	402.52	3.92E-88

If deer within small landscape extents are more likely to contact one another than deer separated by large spatial extents, then including pairs of deer separated by large distances would tend to deflate the average estimates of contact probabilities. To ensure that our results were not being influenced by this effect, we repeated the model selection using datasets including pairs of deer separated by distance from 40 km to just 2 km at 1 km intervals.

### Spatial contact structure

To determine the rate at which contact probabilities decrease with distance across the landscape, we used contact counts per 4 hr time interval to determine the overall direct contact probability of each pairing of individual deer. Mathematically this is accomplished for each deer pairing by dividing the number of direct contact instances by the total number of synchronous positions. This contact probability represents the probability of the pair contacting one another over a given time period, and is fundamentally different than the estimates of daily contact probabilities above.

Individuals in each deer pairing are separated on the landscape by some distance, and this distance changes with time. Of the multiple ways to define the landscape extent separating 2 animals, we used a conservative one: the maximum distance between the observed locations of individual pairs of deer, irrespective of time. This distance choice was based on the practical need for decision makers to evaluate the appropriate landscape extent for management actions and recognizes that individuals in close proximity during a portion of the year may represent potential spread from much greater distances. We calculated this distance for all possible pairings of individuals and then determined how the probability of contact changed with inter-pair distance across the landscape. We sorted all of the pairs by their maximal distance and then accumulated the probability of contact with increasing distance. We then scaled the sum of the contact probabilities over all animals to unity to obtain a cumulative probability distribution as a function of distance. We evaluated the spatial distribution of contact probabilities within spring/summer (May 1-September 30), fall (October 1-December 31), and winter (January 1-April 30) because we expected differing seasonal behaviors to drive the contact processes. Specifically we expected winter migrations and dispersal events to extend the distance across which contacts might occur. Finally, we evaluated how sensitive this measure of spatial structures of direct and indirect contacts is to the cutoff distance we used to define a contact by varying this distance from 25 m to 500 m in 25 m increments.

## Results

**Figure 1:**
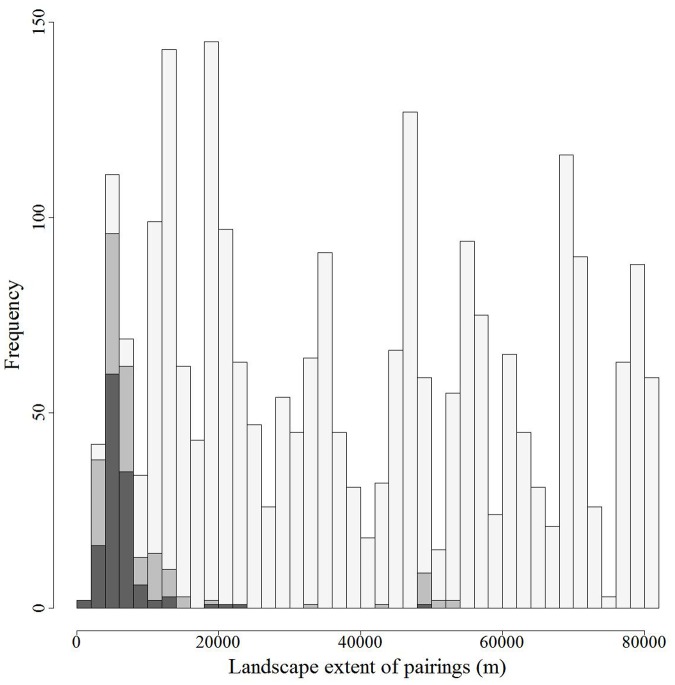
Histograms of landscape extents (m) separating individual pairings of white-tailed deer across central New York State during 2006–2008 for all pairings (light gray, *n* = 2,486), pairings exhibiting indirect contact (medium gray, *n* = 257), and pairings with direct contact (dark gray, *n* = 133). We defined the landscape extent as the maximum observed distance separating each pair irrespective of time.

Of the 2,485 possible unique pairings of the 71 individual deer, we observed potential direct contacts between 125 pairs (5%) of individual deer and indirect contacts between 257 pairs (10%) of deer. The maximum intra-pair distance across the landscape ranged from 942 m to 81,847 m (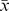 = 39,066 m, SD = 23,730) with good representation of pairs across that range (Figure 1).

### Temporal contact rates

**Figure 2:**
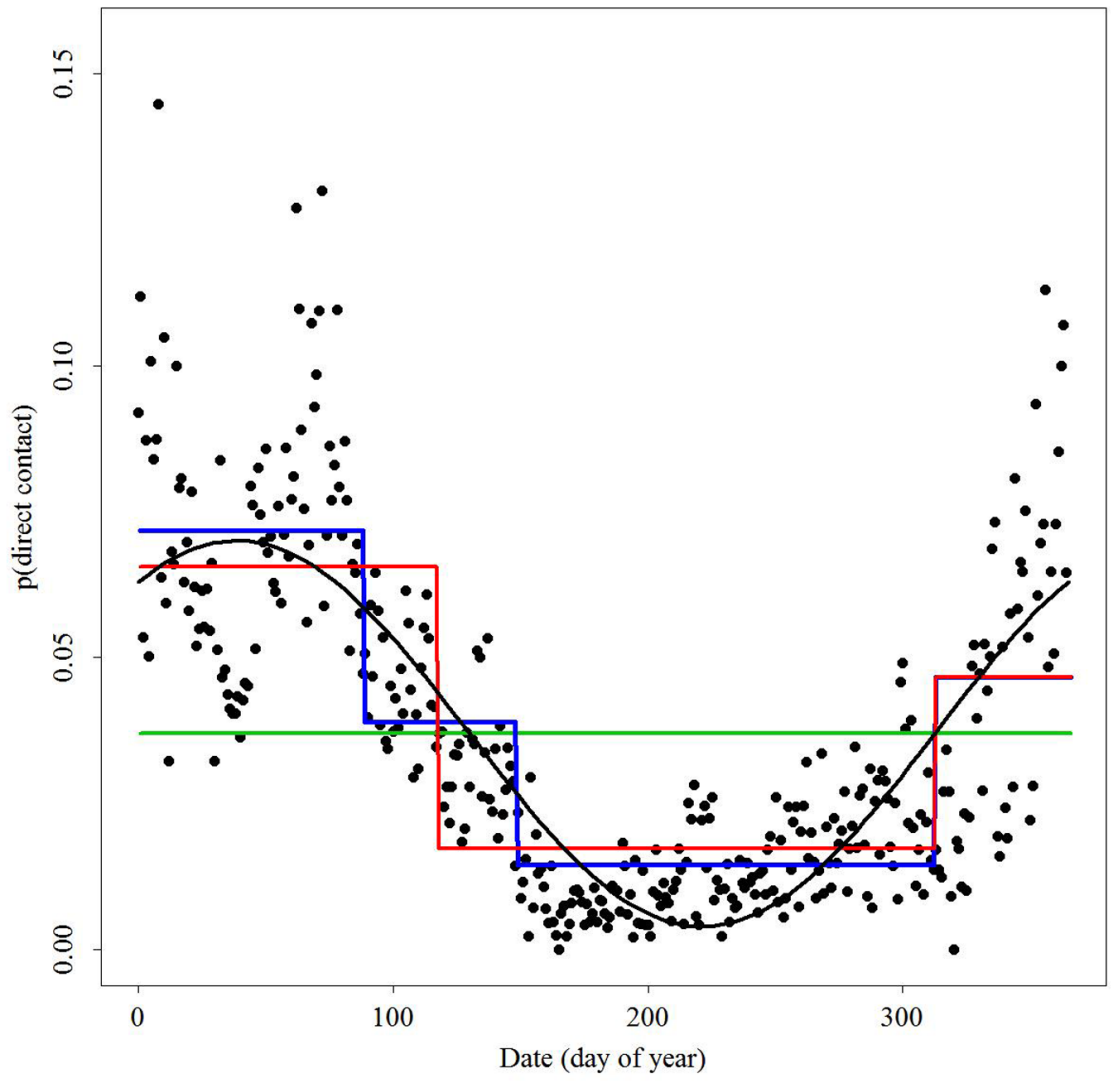
Probability of direct contact among white-tailed deer across central New York State during 2006–2008 as a function of day of year (1 January = 0). Lines represent functions fit to the data: constant contact probability (green), piecewise linear threshold model with 4 break-dates (blue), piecewise linear threshold model with 3 break-dates (red), and a sine function (black).

**Figure 3:**
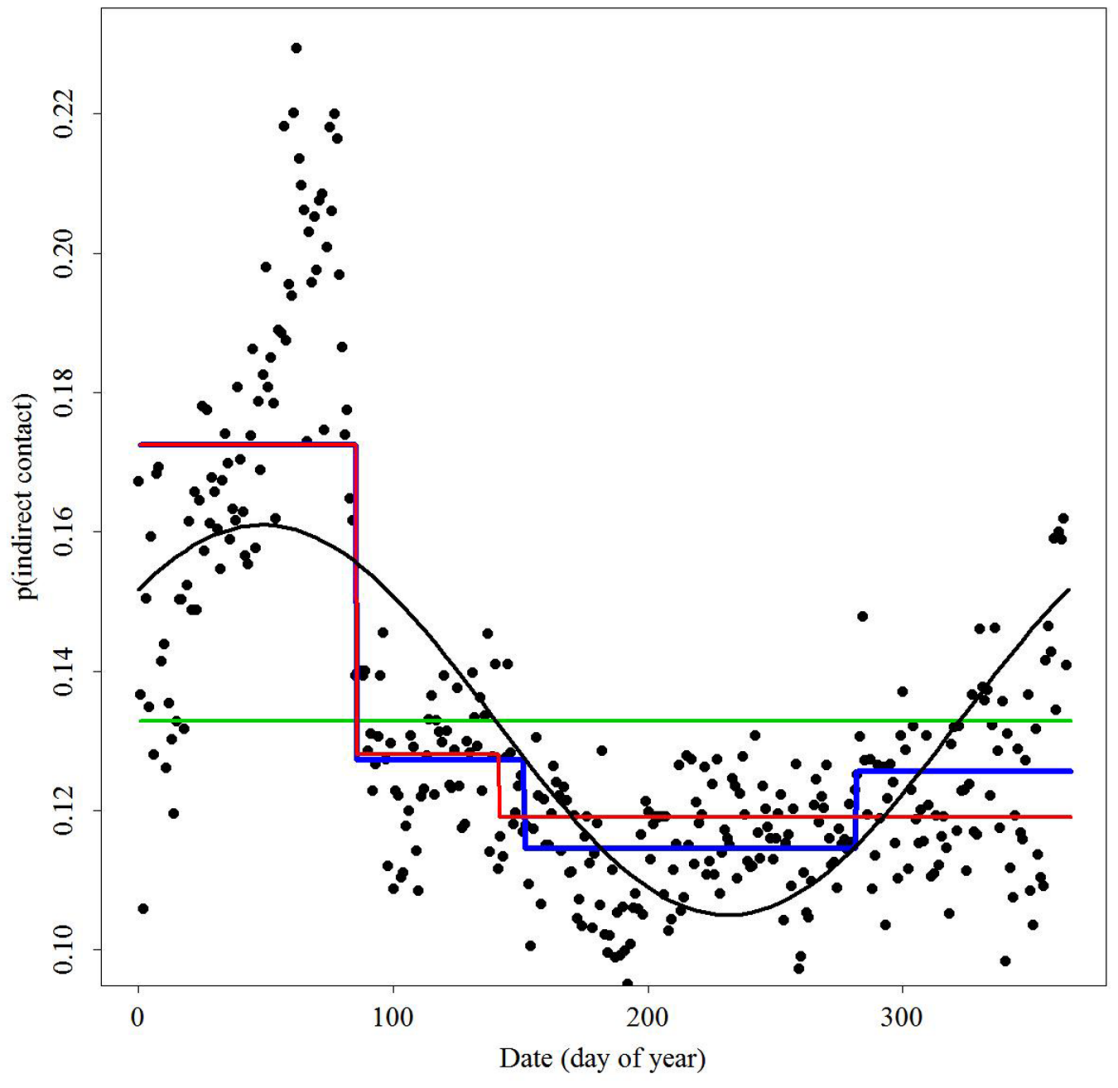
Probability of indirect contact as a function of day of year (1 January = 0) for white-tailed deer in central New York. Lines represent functions fit to the data: constant contact probability (green), piecewise linear threshold model with 4 break-dates (blue), piecewise linear threshold model with 3 break-dates (red), and a sine function (black).

Daily direct contact probabilities varied as a function of time (Figure 2). Daily indirect contact probabilities were nearly 3 times greater than daily direct contact probabilities (Figure 3). We observed the highest and most variable contacts during winter. Daily contact probabilities began declining mid-March and continued to low levels in late May/early June. These values were near zero for direct contact probabilities during that time. Variation in daily contact probabilities began to increase in early October accompanied by an increase in values by late-December. Changes in indirect contact probabilities during spring, summer, and fall were less apparent than those observed for direct contact probabilities over the same seasons.

**Figure 4:**
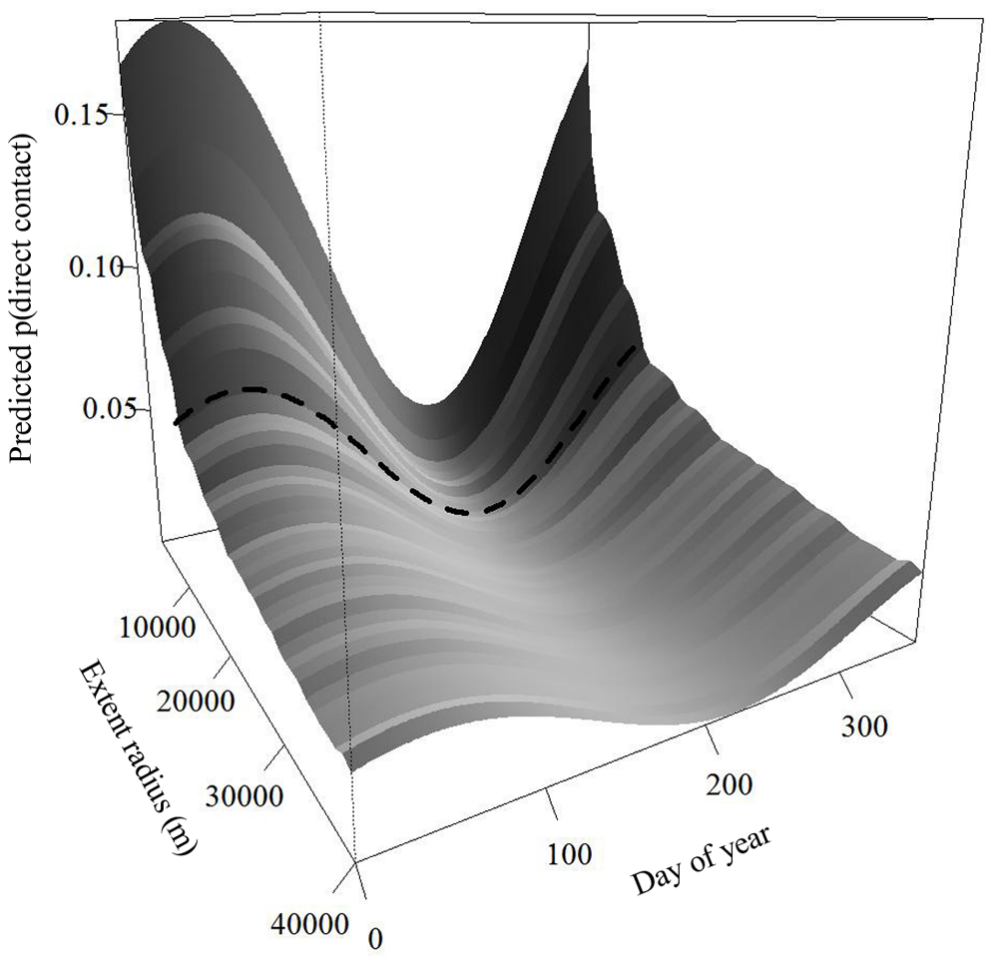
Predicted probability of direct contact among white-tailed deer across central New York State during 2006–2008 as a function of day of year (1 January = 0) and landscape extent within which pairs are included for white-tailed deer in central New York. The dashed line represents the direct contact probabilities for deer within 11,000% of direct contact probabilities.



**Figure 5:**
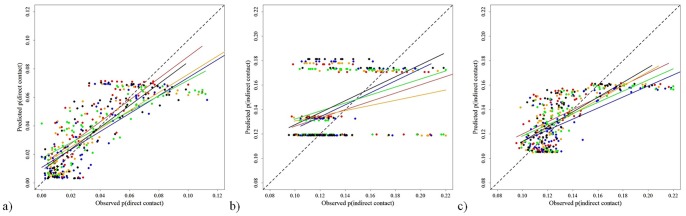
Regressions of observed versus predicted contact probabilities for k-fold cross-validation (k = 5) of models describing probability of contact as a function of time for white-tailed deer in central New York during 2006–2008: a) sine function predicting probability of direct contacts, b) piecewise linear threshold function describing probability of indirect contacts, and c) sine function predicting probability of indirect contacts. Colors distinguish the points and regression lines of each cross-validation fold.

Probability of contact declined as we included individuals from a greater spatial extent and although the intercept and amplitude parameters decreased, the seasonal fluctuations persisted (Figure 4). The best model for the temporal function of direct contact probabilities of deer occurring within 11 km, the distance accounting for 99% of direct contact probabilities, of one another was a sine function:

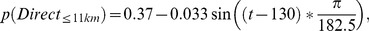
where *p(x)* is the daily probability of contact and *t* is the day of the year (Table 1). The model including an intercept, amplitude, and a phase shift outperformed other models across all examined landscape extents (≤40 km), including the intercept only model. Observed and predicted daily direct contact probabilities were significantly correlated for all folds of the model validation sets (Table 2). Regressions of predicted versus observed contact values for each fold indicated good predictive power for the sine function, with some deviation for the highest observed contact probabilities (Figure 5a). Goodness-of-fit tests indicated that predicted values were not significantly different from observed probabilities for all cross-validation folds except for Fold C (Table 2). The best model for the temporal function of indirect contact probabilities of deer occurring within 11 km of one another was a piecewise linear threshold function:
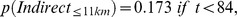


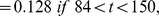


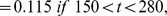


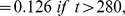
where *p(x)* is the daily probability of contact and *t* is the day of the year (Table 1, blue line). Observed and predicted daily indirect contact probabilities were significantly correlated for all folds of the model validation sets (Table 2). Regressions of predicted versus observed contact values for each fold indicated poor predictive power for the threshold function (Figure 5b). This function frequently underestimated high contact probabilities and overestimated lower contact probabilities resulting in significant differences in goodness-of-fit tests (Table 2). A sine function better predicted indirect contact probabilities based on regression analyses of each fold (Figure 5c). However, the sine function also predicted significantly different bin counts of observed and predicted contact probabilities for all but 1 fold (Table 2).

**Table 2:** Characteristics (rank correlation, regression, and goodness-of-fit) of the accuracy of each fold for 5-fold cross validation of selected models predicting temporal contact probabilities among deer in central New York.

		Rank correlation		Expected vs. observed-regression			χ^2^_(df = 4)_
Model	k-fold	r_s_	p	b_0_(SE)	b_1_(SE)	R^2^	p
*Direct Contact - Temporal*							
	A	0.815	<0.001	0.012 (0.003)^a^	0.762 (0.064)^b^	0.660	0.214*
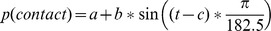	B	0.829	<0.001	0.011 (0.003)^a^	0.629 (0.059)^b^	0.604	0.082*
	C	0.781	<0.001	0.017 (0.002)^a^	0.546 (0.045)^b^	0.671	0.021*
	D	0.822	<0.001	0.009 (0.003)^a^	0.762 (0.060)^b^	0.690	0.053*
	E	0.833	<0.001	0.012 (0.002)^a^	0.637 (0.052)^b^	0.682	0.322*
*Indirect Contact - Temporal*							
	A	0.682	<0.001	0.070 (0.008)^a^	0.497 (0.063)^b^	0.458	0.002*
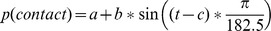	B	0.717	<0.001	0.069 (0.007)^a^	0.452 (0.047)^b^	0.557	0.329*
	C	0.75	<0.001	0.073 (0.008)^a^	0.458 (0.057)^b^	0.467	0.003*
	D	0.689	<0.001	0.054 (0.009)^a^	0.606 (0.064)^b^	0.547	0.001*
	E	0.744	<0.001	0.056 (0.009)^a^	0.573 (0.068)^b^	0.506	<0.001*
threshold model - 4 breakpoints	A	0.448	<0.001	0.092 (0.011)^a^	0.339 (0.080)^b^	0.190	<0.001*
	B	0.441	<0.001	0.075 (0.016)^a^	0.489 (0.117)^b^	0.184	<0.001*
	C	0.378	<0.001	0.099 (0.013)^a^	0.328 (0.095)^b^	0.131	<0.001*
	D	0.429	<0.001	0.079 (0.016)^a^	0.490 (0.122)^b^	0.173	<0.001*
	E	0.243	0.045	0.110 (0.014)^a^	0.210 (0.102)^b^	0.045	<0.001*

^a^ Significantly different from 0.0.

^b^ Significantly different from 1.0.

Significant difference between observed and expected.

### Spatial contact structure

#### Direct Contacts

Non-zero probability of direct contact between paired individuals varied from 0.0006 to 0.86 (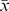 = 0.098, SD = 0.16, *n* = 128). Pairings with some synchronously recorded positions and no observed contact events numbered 1,625, and 732 pairings had no positions recorded during synchronous time intervals (thus no potential for direct contact).

**Figure 6:**
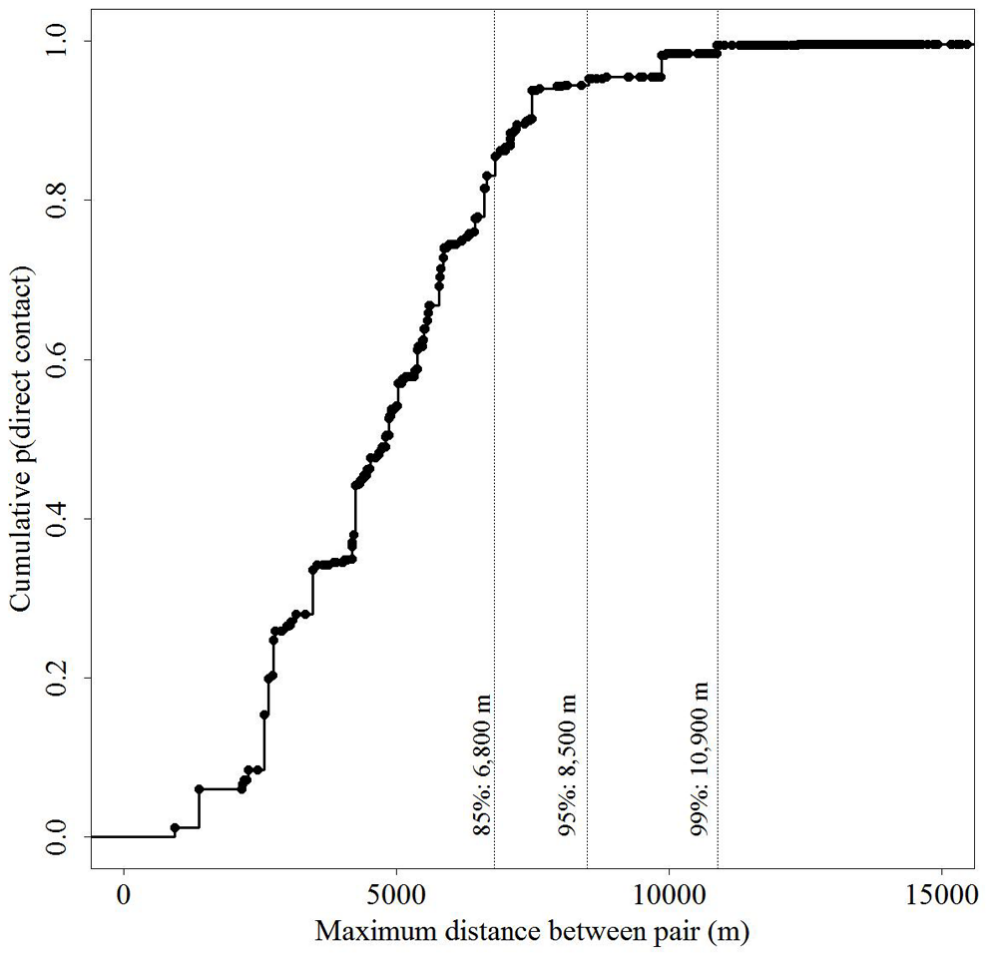
Spatial structure of direct contacts among white-tailed deer in central New York during 2006–2008. Cumulative density function for probabilities of direct contact as a function of maximum observed separation distance (m). Dashed vertical lines indicate the distances at which percentages of direct contact are accumulated.

The cumulative distribution function for the spatial structure of direct contact probabilities increased rapidly with distance from 1,000 m – 7,000 m (Figure 6): 95% of the probabilities of direct contact were accounted for within 8,500 m and 99% within 10,900 m. All probabilities of direct contact were contained within 34,500 m.

**Figure 7:**
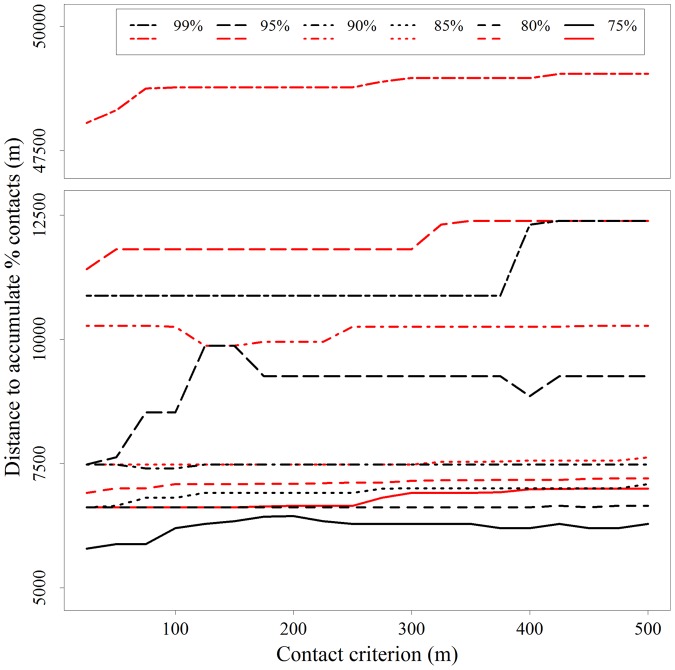
Sensitivity of the spatial structure of direct (black lines) and indirect (red lines) contacts to the distance criteria for defining contacts among white-tailed deer in central New York.

The distance at which probabilities of direct contact were accounted for was not greatly influenced by the cutoff distance used to define potential direct contacts (Figure 7). Varying the distance criterion had little influence on percentages of direct contact for probabilities ≤90%. The distance within which 95% of cumulative probabilities of direct contact occurred increased from 7,500 m to 10,000 m with distance criterion (25–125 m), and then declined to 9,500 m at a distance criteria of 175 m where it remained stable at higher distance criterion values.

**Figure 8:**
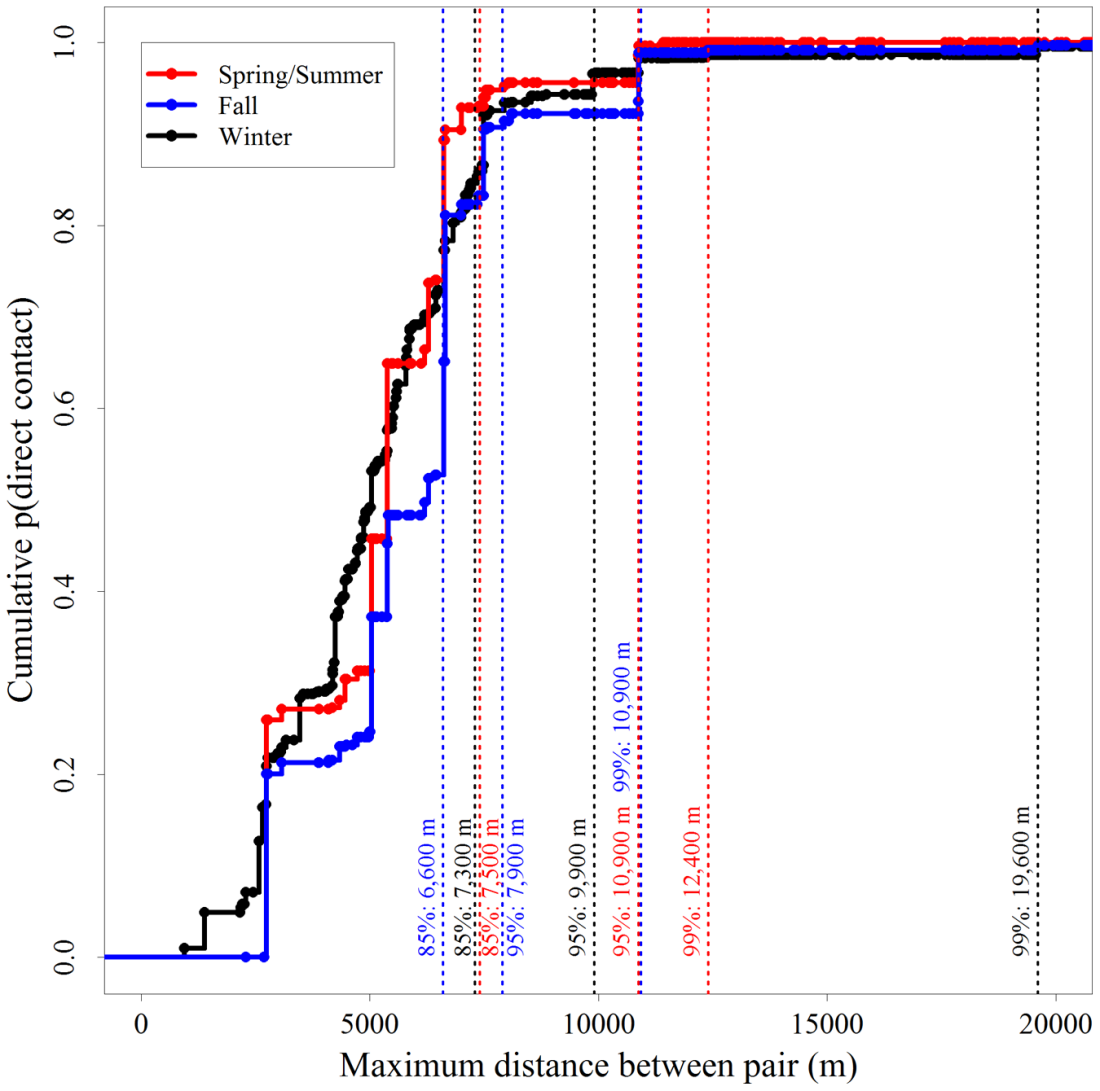
Season-specific spatial structure of direct contacts among white-tailed deer in central New York during 2006–2008. Cumulative density function for probabilities of direct contact as a function of maximum observed separation distance (m). Dashed vertical lines indicate the distances at which percentages of direct contact probabilities are accumulated within each season (spring-summer, fall, and winter).

While there were clear differences in seasonal contact probabilities (Figure 2), the spatial structure of those contacts varied little across seasons (Figure 8). We accounted for 90% of direct contact probabilities within 7,500 m for all seasons. The distance to account for the upper percentages of contact probability differed somewhat. We observed a long tail for the cumulative probability distribution of direct contacts during winter, reflected in the large spatial distance (19,600 m) required to account for 99% of contact probabilities. We observed 99% of direct contact probabilities in the spring/summer and fall to occur within smaller spatial extents (12,400 m and 10,900 m respectively).

#### Indirect contacts

**Figure 9:**
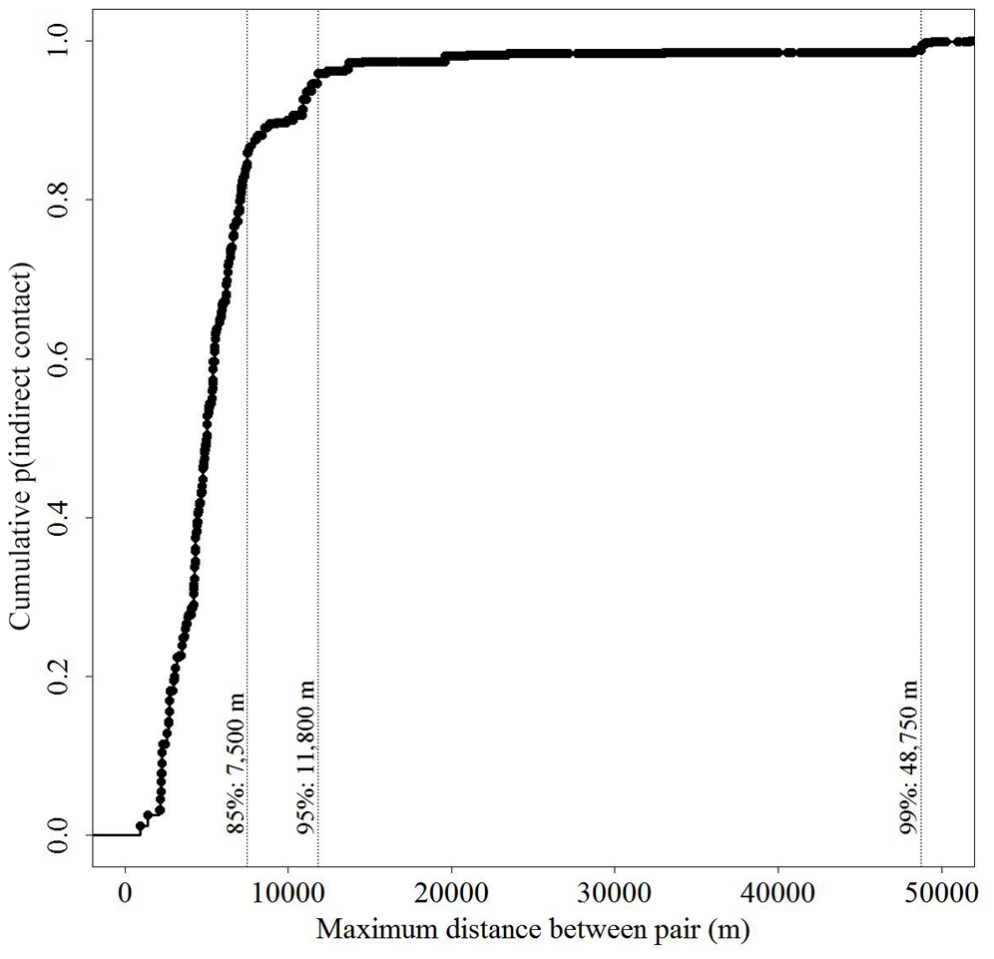
Spatial structure of indirect contacts among white-tailed deer in central New York during 2006–2008. Cumulative density function for probabilities of indirect contact as a function of maximum observed separation distance (m). Dashed vertical lines indicate the distances at which percentages of indirect contact probabilities are accumulated.

Non-zero probability of indirect contact for paired individuals ranged from 0.0003 to 0.97 (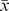 = 0.272, SD = 0.30, *n* = 257). Pairings with the temporal potential for indirect contact that exhibited no indirect contacts numbered 2,228. Probabilities of indirect contact accumulated rapidly in the range 1,000 m to 7,500 m (Figure 9). 95% of the probabilities of direct contact were accounted for within 11,800 m and 99% of contact probabilities were contained at 49,000 m.

The distance at which probabilities of indirect contact were accounted for was not greatly influenced by the cutoff distance used to define potential indirect contacts (Figure 7). The distance within which 95% and 99% of cumulative probabilities of indirect contact occurred increased slightly with distance criterion (25–75 m).

## Discussion

Our study is among the first to provide quantitative estimates of potential contact probabilities that could be used to parameterize transmission in models of disease such as CWD. Our use of large numbers of GPS collars deployed at various distances across a landscape provided the high-resolution movement data necessary to develop a profile of the temporal and spatial structure of contacts between individuals. Although the separation distance we used to define contact events represents potential, but not actual contacts, the distribution of direct contact probabilities was not greatly affected over a wide range of contact criterion.

The temporal pattern of contacts corresponds to the seasonal changes in social behavior of deer. Probabilities of direct contact vary seasonally and are highest in the winter. This observation corresponds with seasonal migration to areas with greater amounts of forested cover, especially of coniferous species, by deer in this region in response to winter conditions. Such migrations by deer are well documented in northern climates and result in bringing deer populations together from across a broad landscape [10]. The probability of direct contact among individuals peaks in March, decreases rapidly in early spring as individuals return to summer range, and is lowest during late May and early June when females isolate themselves for parturition (Figure 2) [11], [32], [33]. Direct contact probabilities remain low until fall when they increase in association with rut in November and December. These seasonal patterns in contact probability persisted, though at decreasing levels, even when we included individuals separated by great distances (Figure 4). These seasonal dynamics in contacts were reasonably represented by a sine function, although predictions were inaccurate during the winter when observed contact probabilities were high (Figures 2 and 5a).

The importance of documenting this temporal dynamic in direct contact structure is threefold. First, the fact that observed changes in contact probabilities correspond to well-documented changes in seasonal behavior among white-tailed deer suggests that we are defining contacts appropriately and that contact rates scale with density. Second the predictability of the seasonal dynamics of contact is valuable because season-specific probabilities of contact can be reasonably described as a sine function, which is easily integrated into the transmission parameter, β, of disease models. With additional information on the probability of contacts resulting in infection, such models can be used to evaluate the potential number of new infections per time interval. Third, one can estimate how the spatial structure of deer populations influence those contact probabilities and incorporate appropriate functions describing contact probability for a population occupying a certain landscape extent (Figure 4).

Similar to the temporal dynamics of direct contact, the probability of indirect contact was highest during winter, peaking in March (Figure 3). The peak in March is likely a result of individuals that are using different areas of the landscape in the summer and fall but using communal wintering areas. During the rest of the year probability of indirect contact was generally constant at levels comparable to high observed daily values for direct contact of deer within 11 km of one another (0.10 – 0.13). This difference in direct and indirect contact probabilities indicates that while areas of space use among deer overlap, those individuals are not spending proportional amounts of time in direct contact with one another (<100 m). Deer in an area may be using the same locations on the landscape but appear to directly interact less than expected, at least during those periods we could observe with our collars. Because indirect contacts are identifying contact with space previously visited by another individual over a long period of time, probabilities of indirect contact are expected to be greater in value, but fluctuate less than those associated with direct contact. This expectation was supported by the piecewise-linear threshold model providing the best fit to the indirect temporal contact probabilities, where those probabilities varied only slightly during seasons other than winter. These results suggest that indirect contacts may be less variable throughout the year in regions where deer do not use communal areas during winter.

The sine functions we used are symmetrical and constrained by an annual period and we did not attempt to model dynamics of seasonally partitioned contact probabilities. Consequently, we could not identify differences between contact dynamics during only a portion of the year. The potential for differences in amplitudes of fluctuation may be compounded by our binomial classification of indirect contacts (a deer was present at a location or not), which does not account for the number of times a location was previously visited. Our binomial approach may be insufficient to characterize the degree of contamination of a location and thus may have important implications to the probability of disease transmission because repeated visits to a site by infected individuals would increase the quantity of disease agent shed. However, we opted for a parsimonious approach because little is known about infectious doses required for environmental transmission of CWD [24]. If individuals are seasonally visiting locations that were frequently used in the past, then transmission may fluctuate seasonally in ways not identified solely by our indirect contact function.

Quantifying the spatial structure of contacts as a function of distance informs another aspect of disease risk models by providing an explicit probability distribution for disease spread across a landscape. White et al. [34] demonstrated this relationship with rabbits, showing declining interactions between rabbit warrens as a function of distance, although the authors did not then attempt to quantify the percentage of contacts occurring within a certain distance. Our findings show that the risk surface depicting the probability of direct contact drops rapidly with distance from a point of occurrence and that 90% of contact probabilities are likely to occur within 7 km. The cumulative probability of contact reaches 99% within 11 km (Figure 6). These distances are half and three-quarters of the radius that was used to delineate a containment area within New York State (16 km).

The spatial extent of direct contact probabilities is consistent across seasons despite the variation in temporal patterns of contact. This was surprising because deer exhibit season-specific behaviors. It appears that despite seasonal changes in behavior and probabilities of direct contact, the contacts that do occur within each season are distributed proportionally across similar spatial extents (Figure 8). The primary difference between seasons is the long tail for winter revealing that a small percentage of contacts in winter occur between animals separated by large distances during the rest of the year. This tail likely represents seasonal migrations of deer that are widely separated during the summer, but encounter one another within traditional wintering yards. Alternatively, the tail may reflect animals that dispersed during the spring or fall and established new home ranges away from their natal ranges [35], [36].

We used the probabilities of contact between each deer pair to plot the accumulation of direct contacts over space versus the maximum separation distance based on all observed locations between paired individuals irrespective of time (including season). This distance choice was based on the practical need for decision makers to evaluate the appropriate landscape extent for management actions within a season, but recognizes that individuals in close proximity during a portion of the year may represent potential spread from much greater distances. For example, if we neglected to incorporate landscape extents represented throughout the year and only evaluated the spatial structure of contacts in the winter we would likely conclude that a majority of contacts occurred across a very small landscape extent (on the scale of wintering areas).

A much greater spatial extent is required to account for the last few percentage points of the cumulative probability of indirect contact than direct contacts. Like the temporal patterns, this difference in spatial probabilities is driven by the long time interval during which CWD prions may persist in the environment. This interval allows migrating and dispersing animals from multiple generations to encounter infected individuals indirectly. Current research suggests that prions can persist in the soil, especially those with high clay content, for at least 2 years [24], [37]. While the probability of indirect contact is most likely to occur within 12 km, the 99% cumulative probability extends out to 49 km (Figure 9). The more distant events represent a relatively small portion of indirect contacts across space, but models that employ a more conservative criterion for evaluating risk of infection need to be spatially extensive.

Implicit to our analyses are 4 principal assumptions about how we defined direct and indirect contacts pertinent to CWD when using data from GPS collars. First, our temporal analysis assumes that all deer ≤11 km apart have the potential to experience direct or indirect contact daily. The obvious question is how to scale the analysis to include the appropriate geographic extent because the farther apart individuals are from one another the less likely they are to contact one another. This becomes analytically challenging because including contact data on animals separated by great distances adds many zeros and decreases resulting daily contact probabilities. We chose not to include individuals separated by >11 km because this was the distance that accounted for 99% of direct contact probabilities.

Second, our analysis of the spatial structure of direct contact could be dependent on the criterion by which we identified potential contacts among GPS locations. Previous studies have used separation distance between GPS or radio telemetry locations to identify contact between individuals [12], [20], [21]. These distances are generally based upon an understanding of the movement potential within the sampled time intervals for the organism in question and the positional error for location acquisition. When more specific information about the capacity for disease transfer is known (e.g., the distance an aerosol pathogen such as tuberculosis may travel), those distances are more easily defined [16]. We defined contacts as synchronous locations that occurred within 100 m. We evaluated the sensitivity of the spatial structure of direct and indirect contact probabilities to our definition by testing a range of potential distances by which contacts may be defined. We found most percentiles of cumulative contact probability to not be influenced by the contact criterion. This consistency suggests that the separation distance between deer pairs scales proportionally across all pairings for the range evaluated (25 m to 500 m). However, we did find that the distance at which 95% of direct contacts were accounted was sensitive to the contact criterion. This may be important as 95% is a percentile consistently, though arbitrarily, chosen to describe distributions.

Daily contact probabilities are likely to be impacted by changes in contact criterion. We have not formally evaluated the impact of such changes, but have several expectations for the influence of the contact criterion on daily contact probabilities. If the distances separating deer at any point in time are uniformly distributed, we would expect the daily contact probability function to shift upwards or downwards with increasing or decreasing contact criterion distance. For example if we decrease the distance required to identify a contact from 100 m to 50 m, we would expect to identify fewer contact events in the set of observed events. This adjustment would decrease the probability of contact for any given day. Because contact probabilities are bounded on the lower end by zero, we would not only expect changes in the intercept of a function describing contact probabilities over time, but in the amplitude of that function with decreasing contact criterion distances.

Third, using contact events to characterize contact probabilities for the population across time and space assumes our sampling adequately represents the population of contact probabilities across time and space. The question of whether we are accurately representing the probability of contact for individuals in a population based on a sample of paired individuals has been discussed as a limitation to such studies [12]. Arguably the accuracy of these probability-of-contact values depends upon whether the collared animals adequately represented the population of contacts. One possibility is that we collared an insufficient number of individuals to characterize the relationship. The suggestion that collaring more individuals might change the results implies that we have over- or under- represented some important component of the population with respect to contacts. We attempted to sample individuals across many different spatial extents (Figure 1) and our findings suggest that important information may be gathered and contact probabilities quantified using a smaller percentage of the population than has previously been implied.

Finally, the assumption that uninfected individuals will behave, move, and contact one another in manners similar to diseased individuals is an important one. While this assumption is probably valid until late stages of CWD, we were not able to evaluate this potential discrepancy and acknowledge that differences in movement by diseased animals may alter the temporal and spatial contact structures among individuals as quantified in our study.

## Conclusions

Our results have direct application for those attempting to model diseases such as CWD among white-tailed deer. The equations describing direct and indirect contact as a function of time substitute directly into the portion of the force of infection describing the probability of contact. Consider the force of infection for direct contact assuming frequency dependence,



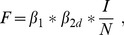
where *β_1_* is the probability of infection given a contact, *β_2d_* is the probability of a direct contact, and *I/N* is the proportion of infectious individuals in the population. From our data we generated the seasonally forced function for probability of direct contact for a population of deer living within a 380 km^2^ area (defined by an 11 km radius),



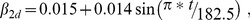
where *t* represents the day of the year. Substitution results in a refined and empirically informed force of infection for direct contacts,



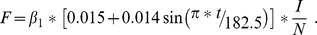



Similar substitution applies for indirect contact and the combination of the 2 contact processes. Quantifying seasonal changes in contact probabilities among deer so that they may be incorporated into epidemiological models is an important step towards empirically informing unknown parameters.

While our results have quite specific applications to modeling disease among white-tailed deer, more generally our findings show that contacts vary seasonally in a manner consistent with expectations based upon known behaviors. While behavioral information alone cannot specify the magnitude of contact probabilities, those working with other species and systems may have justification for developing the form of contact functions for modeling purposes. We also suggest that these functions may be determined without monitoring every member of a population.

Our findings also indicate an important shift in how we approach managing risk and assessing feasibility of management actions. There exists an intuitive understanding that the more separated individuals are on the landscape the less likely they are to come into contact with one another. Unfortunately, the specifics of this function are generally unknown and, without empirical data on contacts between individuals across a landscape, cannot be quantified. So, given a point of occurrence, managers must assume a uniform distribution of contacts across some extent. The maximum potential extent of this relationship may be informed by the maximum movement potential of the species in question. However such information typically incorporates extreme or atypical movements. While such movements may be important to disease spread, for large mammals the spatial extent represented by these extremes quickly exceeds a landscape extent that may be feasibly managed. Indeed, we found that the spatial extents (radii) from a point of occurrence required to account for all observed contact events (direct = 48.7 km, indirect = 52.2 km) are likely too great to effectively manage. However, by quantifying the distribution of contact probabilities across space we observed that a large percentage of those contact probabilities occurred at much smaller extents.

Equipped with such a distribution of contacts across space, managers can make decisions such as what landscape extent must be considered in order to account for a certain percentage of contacts, or what percentage of contacts are accounted for given limitations for what landscape extent can be realistically managed. This distribution of contacts may be coupled with other information such as resource selection functions to refine probability surfaces for identifying risk for disease spread. We suggest that describing spatial extents of management actions based upon empirically informed probabilistic surfaces of risk is the logical next step in the approach to disease management. Our distributions of spatial contact structures are a new and important component of those surfaces.
